# Comparative Analysis of Demographic and Clinical Findings in Spontaneous, Peritoneal Dialysis-Related, and Secondary Bacterial Peritonitis

**DOI:** 10.7759/cureus.55445

**Published:** 2024-03-03

**Authors:** Tülay Unver Ulusoy, Hanife Nur Karakoç Parlayan, Nilgün Altın, Büşra Sarıkaya, Büşra Öztürk, İrfan Şencan

**Affiliations:** 1 Infectious Diseases and Clinical Microbiology, Ankara Etlik City Hospital, Ankara, TUR; 2 Infectious Diseases and Clinical Microbiology, Health Sciences University Dışkapı Yıldırım Beyazıt Training and Research Hospital, Ankara, TUR

**Keywords:** peritonitis treatment, secondary peritonitis, peritoneal dialysis-related peritonitis, spontaneous bacterial peritonitis, peritonitis

## Abstract

Background

This study aims to contribute to peritonitis management strategies by comparing the demographic, clinical, and laboratory characteristics of patients diagnosed with spontaneous bacterial peritonitis (SBP), peritoneal dialysis-related peritonitis (PDrP), and secondary peritonitis.

Methods

This study included 86 patients diagnosed with peritonitis between 2016 and 2022. Patients were categorized and compared as SBP, PDrP, and secondary peritonitis.

Results

SBP was diagnosed in 36% of patients, secondary peritonitis in 36% and PDrP in 28%. The mean age of patients with PDrP is 43.71 ± 14.74, which is significantly lower compared to those with SBP and secondary peritonitis (p<0.001). Patients with hypertension (HT), chronic kidney disease (CKD), and those undergoing dialysis most commonly have PDrP whereas those without HT, without CKD, and not undergoing dialysis are most often diagnosed with secondary peritonitis (p=0.002, p<0.001, p<0.001). In peritoneal fluid cultures, the growth of Gram-positive bacteria was most commonly identified in patients with PDrP, while the growth of Gram-negative bacteria was most frequently seen in patients with secondary peritonitis (p=0.018). CRP levels and sedimentation rates were found to be higher in patients with secondary peritonitis (p<0.001, p=0.003).

Conclusion

The distinct characteristics observed across different types of peritonitis underscore the importance of tailored approaches to diagnosis and treatment. Parameters such as CRP levels, sedimentation rates, and patient age could serve as valuable indicators in discerning between various types of peritonitis. When selecting empirical antibiotic therapy, it's crucial to consider coverage for Gram-positive pathogens in cases of PDrP and Gram-negative pathogens in secondary peritonitis.

## Introduction

The peritoneum, a protective serous membrane in the abdominal cavity, serves as the body's initial barrier against infections [1]. Peritonitis, the membrane inflammation, is categorized into primary and secondary forms [2]. Primary peritonitis occurs when bacteria infiltrate the peritoneal cavity through translocation, spread via the bloodstream, or unintentional medical introduction, all without any apparent physical breach in the gastrointestinal system [3]. This category of peritonitis encompasses spontaneous bacterial peritonitis (SBP) and peritoneal dialysis-related peritonitis (PDrP) [4,5]. Although SBP and PDrP are categorized as primary peritonitis, the patient population affected, causative pathogens and treatment approaches are dissimilar [2]. SBP is a grave and common complication among patients with liver cirrhosis and ascites, arising primarily from the migration of bacteria, often from the intestinal tract, into the ascitic fluid [5]. Peritoneal dialysis (PD) is a widely used renal replacement method for managing end-stage renal disease (ESRD), utilizing the peritoneal membrane as a dialyzing surface. PDrP typically occurs due to non-aseptic techniques or contamination associated with the PD catheter or its connections [4]. In contrast, secondary peritonitis is caused by the direct invasion of the peritoneum due to leakage from the gastrointestinal or urogenital tracts, or from adjacent solid organs [6]. The mortality associated with peritonitis is often due to systemic inflammation and sepsis. Consequently, prompt and appropriate antibiotic treatment is fundamental in managing bacterial peritonitis [2]. Although SBP, PDrP, and secondary peritonitis are infections of the same organ, their pathogeneses are distinct, leading to differences in the affected patient populations, causative pathogens, immune responses, treatment algorithms, and disease courses [2,4,5,7,8]. Previous studies have evaluated the demographic characteristics, and clinical, and laboratory findings of patients with SBP, PDrP, and secondary peritonitis. However, research comparing patients across these types of peritonitis is quite limited [8-10]. Understanding the differences according to the type of peritonitis can aid in identifying patients at high risk and assist in the development of early diagnosis, appropriate antibiotic therapy, and monitoring strategies. This study aims to contribute to peritonitis management strategies by comparing the demographic, clinical, and laboratory characteristics of patients diagnosed with SBP, PDrP, and secondary peritonitis.

## Materials and methods

This study included patients diagnosed with peritonitis between 2016 and 2022 at the Health Sciences University Dışkapı Yıldırım Beyazıt Training and Research Hospital, Ankara. Inclusion criteria were: diagnosis of peritonitis made at the hospital, inpatient care, admission or consultation in the Infectious Diseases and Clinical Microbiology (IDCM) clinic, antibiotic therapy managed by the IDCM clinic, and being ≥ 18 years of age. Exclusion criteria included: transfer to another hospital or discharge after diagnosis, exclusion of the diagnosis after initiating peritonitis treatment, diagnosis of peritonitis types other than SBP, PDrP, and secondary peritonitis, and being under 18 years of age.

Patients were categorized into SBP, PDrP, and secondary peritonitis. A diagnosis of PDrP was made in patients with a peritoneal dialysis catheter if at least two of the following criteria were met: (1) clinical signs indicative of peritonitis, such as abdominal pain and/or cloudy dialysis effluent; (2) dialysis effluent white cell count > 100/mL or > 0.1 x 10^9/L (after a dwell time of at least 2 hours), with > 50% polymorphonuclear leukocytes (PMN); and (3) positive culture from dialysis effluent [4].

The diagnosis of SBP is accepted as an ascitic fluid PMN count > 250/µl, along with the exclusion of secondary causes of peritonitis and malignant ascites [5]. Acute peritonitis resulting from the disruption of the integrity of the gastrointestinal tract or genitourinary organs due to an intra-abdominal pathology and subsequent microbial contamination is considered secondary peritonitis [11].

Blood cultures were obtained and analyzed for both aerobic and anaerobic bacteria. The outcomes of these cultures were utilized to inform the selection of empirical antimicrobial therapies, tailored according to the peritonitis category, underlying risk factors, and anticipated pathogens. Survival status was determined based on discharge status, with individuals discharged being classified as survivors. Conversely, patients who died within one month following diagnosis or whilst hospitalized were categorized as non-survivors.

Organization and analysis of the research data

Demographic variables (such as age, gender, comorbidities, dialysis status, admitting department, and intensive care unit admissions) along with laboratory findings (including leukocyte count, neutrophil percentage, and erythrocyte count in peritoneal fluid, as well as white blood cell count, neutrophil count, C-reactive protein (CRP) levels, erythrocyte sedimentation rate, and procalcitonin levels in blood) were reviewed at diagnosis for all patients. The study data were collected retrospectively from the hospital's information management system. This study has received ethical approval from the Ankara Etlik City Hospital Ethics Committee (approval no: AESH-BADEK-2024-053, approval date: January 31, 2024). All statistical analyses were performed using the statistical software Statistical Package for Social Sciences (SPSS), version 22.0 (IBM Corp., Armonk, NY, USA). Data were reported as counts (n) and percentages (%) for categorical variables, and means ± standard deviations for continuous variables. The categorical variables were analyzed using the Chi-square test or Fisher's exact test. To compare continuous variables between SBP, PDrP, and secondary peritonitis groups, The ANOVA (Analysis of Variance) test or Kruskal-Wallis H test was used based on adherence to statistical assumptions. A p-value of less than 0.05 was considered statistically significant for all tests.

## Results

A total of 86 patients with peritonitis were included in the study. Of the patients, 31 (36%) were diagnosed with SBP, 31 (36%) with secondary peritonitis, and 24 (28%) with PDrP (Table 1). The mean age of patients with PDrP is 43.7 ± 14.7, which is significantly lower compared to those with SBP (64 ± 13) and secondary peritonitis (61.5±17.9) (p<0.001). The diagnosis of SBP was recorded in 25 (44.6%; 95% CI: 33.4%-57.6%) men and PDrP in 15 (50%;95% CI: 33.2%-66.8%) women. SBP was the most common diagnosis among men, while PDrP was most frequent among women (p=0.003). Comorbidities were present in 81.3% of patients. Patients with hypertension (HT) (17 {70.8%; CI: 50.8%-85%}), those with chronic kidney disease (CKD) (24 {100%; 95% CI: 86.2%-100%}), and those undergoing dialysis (24 {100%; 95% CI: 86.2%-100%}) were most frequently diagnosed with PDrP. In contrast, patients without HT (24 {77.4%; 95% CI: 60.2%-88.6}), without CKD (29 {93.5%; 95% CI: 79.2%-98.2}), and not undergoing dialysis (29 {93.5%; 95% CI: 79.2%-98.2}) were most commonly diagnosed with secondary peritonitis (p=0.002, p<0.001, p<0.001). All patients with SBP had a diagnosis of liver cirrhosis and presented with ascites. No significant difference was found between the rates of ICU admission and fatality among the patients (p=0.055, p=0.199).

**Table 1:** Demographic and clinical characteristics of patients with peritonitis. Results were expressed as mean ± standard deviation or as a number (n) and percentage (%). The ANOVA (Analysis of Variance) test and Chi-square test (or Fisher’s exact test when appropriate) were employed. A p-value of less than 0.05 was considered to indicate statistical significance. PD: Peritoneal dialysis.

	Total n (%)	Spontaneous bacterial peritonitis n (%)	PD–related peritonitis n (%)	Secondary peritonitis n (%)	p-value
	n:86 (100)	n:31 (36)	n:24 (28)	n:31 (36)	
Age (mean ± ss)	57.4 ± 17.5	64 ± 13	43.7 ± 14.7	61.5±17.9	p<0.001
Female	30 (34,9)	6 (7)	15 (17,4)	9 (10,5)	0.003
Chronic renal failure	34 (39.5)	8 (9.3)	24 (27.9)	2 (2.3)	p<0.001
Diabetes mellitus	17 (19,8)	10 (11,6)	4 (4,7)	3 (3,5)	0.090
Heart failure	11 (12.8)	7 (8.1)	1 (1.2)	3 (3,5)	0.161
Hypertension	37 (43.0)	13 (15,1)	17 (19,8)	7 (8,1)	0.002
Coronary artery disease	10 (11,6)	6 (7)	2 (2,3)	2 (2,3)	0.331
Presence of dialysis	32 (37.2)	6 (7)	24 (27.9)	2 (2.3)	p<0.001
ICU admission	24 (27.9)	8 (9.3)	3 (3.5)	13 (15.1)	0.055
Outcome					
Survive	68 (79.1)	21(24.4)	21 (24.4)	26 (30.2)	0.199
Non-survive	18 (20.9)	10 (11.6)	3 (3.5)	5 (5.8)	

**Figure 1:**
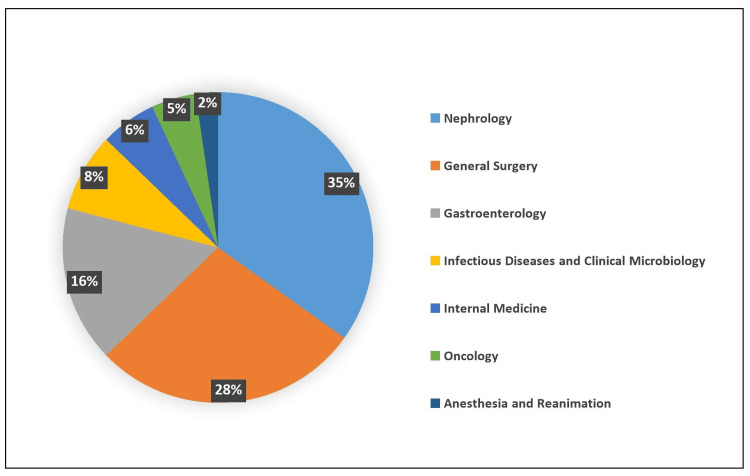
Departments for patients hospitalized with peritonitis.

Patients were most frequently managed in the nephrology clinic (39%), followed by the general surgery clinic (31%) (Figure 1). Patients with SBP were primarily seen in the gastroenterology clinic (35.4%), those with PDrP were exclusively in the nephrology clinic, and patients with secondary peritonitis were most commonly followed in the general surgery clinic (74.1%).

**Figure 2:**
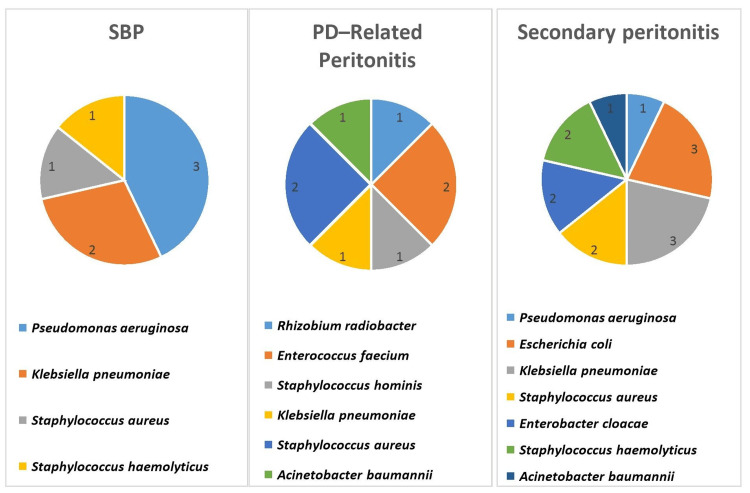
Bacteria isolated in peritoneal fluid based on the types of peritonitis.

Blood cultures were taken from 82 (95.3%) of the patients, with 76 (92.6%) showing no growth. Growth was detected in the blood cultures of four patients with secondary bacterial peritonitis and two patients with SBP. *Escherichia coli* was identified in two patients, Enterobacter cloacae in two patients, *Enterococcus faecium* in one patient, and *Klebsiella pneumoniae* in one patient. Peritoneal fluid cultures were obtained from 80 (93%) of the patients, with growth detected in 30 (34.8%) cases. Among peritoneal fluid cultures showing Gram-negative growth; 12 (60%) were diagnosed with secondary bacterial peritonitis, 5 (25%) with SBP, and 3 (15%) with PDrP. Similarly, in cases with Gram-positive bacterial growth, 7 (70%) were identified with PDrP, 2 (20%) with secondary peritonitis, and 1 (10%) with SBP. The growth of Gram-positive bacteria was most commonly identified in patients with PDrP (7 (70%; 95% CI: 39.7%-89.2%)), while the growth of Gram-negative bacteria was most frequently seen in patients with secondary peritonitis (12 (85.7%; 95% CI: 60%-96%)) (p=0.018). All bacterial cultures from peritoneal fluid showed monobacterial growth. The causative agents grown in peritoneal fluid cultures, differentiated by types of peritonitis, are displayed in Figure 2.

In Table 2, the blood and peritoneal fluid laboratory findings of the patients are examined. The results for CRP and sedimentation rate show significant differences among the types of peritonitis (p<0.001, p=0.003). In secondary peritonitis, both the CRP level and the sedimentation rate are significantly higher compared to those in SBP and PDrP.

**Table 2:** Evaluation of laboratory findings in patients with peritonitis. Results were expressed as mean ± standard deviation. According to distribution The ANOVA (Analysis of Variance) test or Kruskal-Wallis H test was used. A p-value of less than 0.05 was considered to indicate statistical significance. PD: Peritoneal dialysis; SBP: Spontaneous bacterial peritonitis.

	Total n (%)	SBP n (%)	PD–related peritonitis n (%)	Secondary peritonitis n (%)	p-value
Peritoneal fluid laboratory findings (mean ± ss)					
	n:53 (100)	n:29 (54.7)	n:20 (37.7)	n:4 (7.6)	
Leukocyte	4735±9662	6747.93 ±12590	2645±2740	594±288	0.235
Percentage of neutrophils	83.3±7.0	84.6±7.1	82.0±7.14	80.0±4.0	0.275
Erythrocyte	1448±4279	2590.8±5568	58±92	120±240	0.101
Blood test findings (mean ± ss)					
	n:86 (100)	n:31 (36)	n:24 (27,9)	n:31 (36)	
Leukocyte	12650.6±7392	13134 ±8962	10513.88±5708	13821.2±6649	0.235
Neutrophil	10010±6498	9411±7578	8739±5403	11592±5964	0.223
C-reactive protein	168±123	114±109	153±118	233±113	p<0.001
Sedimentation	49.7±21.6	40±22	50.7±19	58.3±19.2	0.003
Procalcitonin	7.6±17.1	6.1±18.4	12.6±23.1	5.2±7.4	0.237

Of the patients, 24 (27.9%) were on peritoneal dialysis, and eight (9.3%) were undergoing hemodialysis. Seventy (81.3%) of the patients received treatment via intravenous (IV) route. In comparison, 16 (18.7%) received intraperitoneal (IP) treatment, and there is a significant difference in the distribution of treatment types according to the type of peritonitis (p<0.001). Patients receiving IV antibiotic therapy were diagnosed 30 (42.9%) with SBP, 30 (42.9%) with secondary peritonitis, and 10 (14.3%) with PDrP. Among those given IP antibiotic therapy, 14 (87.5%) had PDrP, while 1 (12.5%) were diagnosed with secondary peritonitis. The empirical treatment most commonly used was ceftriaxone+metronidazole in 12 (38.7%) patients for SBP, IP cefazolin+amikacin in 11 (45.8%) patients for PDrP and piperacillin-tazobactam in 11 (35.4%) patients for secondary peritonitis.

## Discussion

Bacterial peritonitis is a rare but life-threatening infection, and this study compares patient groups with SBP, PDrP, and secondary peritonitis. Patients with PDrP tend to be younger on average. Women are most frequently in the PDrP group, while men are predominantly in the SBP group. Patients with HT, CKD, and those undergoing dialysis most commonly have PDrP, whereas those without HT, without CKD, and not undergoing dialysis are most often diagnosed with secondary peritonitis. In peritoneal fluid cultures, the growth of Gram-positive bacteria was most commonly identified in patients with PDrP, whereas the growth of Gram-negative bacteria was most frequently seen in patients with secondary peritonitis. The levels of CRP and the rate of erythrocyte sedimentation were found to be higher in patients with secondary peritonitis compared to those with other types of peritonitis.

PDrP is the most concerning PD-related infection as it carries significant morbidity, mortality, and added treatment costs [7]. In our study, 28% of the patients had PDrP, with a mean age of 43.7 ± 14.7, which was younger compared to other groups. A study involving 7051 patients across seven countries, which investigated the peritonitis rate, found that the average age of individuals undergoing PD ranged between 58 and 64 [10]. In a study that included 181 patients who developed PDrP, the average age was reported as 63 ± 17, while another study involving 96 individuals with PDrP reported an average age of 47.9 ± 16.4 [12,13]. Patients who require dialysis due to end-stage renal disease and are capable of performing peritoneal dialysis themselves, being trained in infection control measures, and being able to adhere to recommendations, are administered PD [14]. The lower mean age observed in patients with PDrP in our study might be linked to a preference for placing PD catheters in younger individuals. Additionally, the higher mean age found in patients with SBP or secondary peritonitis, often associated with gastrointestinal perforation, suggests a correlation with severe liver disease, a common finding in our study.

In our study, a notable finding was that the majority of patients in the PDrP group were women, comprising 62.5% of the group, which was significantly higher compared to other peritonitis groups. This contrasts with previous literature, which predominantly reported PDrP patients as being male [7,10,12,15].

In a study presenting data from 391 patients undergoing PD over 10 years, it was reported that 55.2% of those who developed PDrP were women. In a study analyzing data from 352 patients undergoing PD over 12 years, it was noted that 74 patients (20.7%) encountered their first instance of peritonitis within the initial six months. Interestingly, the subset with early-onset peritonitis exhibited a higher representation of female patients [16]. Conducting more extensive studies specifically examining gender distribution among PDrP patients could offer valuable guidance.

It is known that secondary peritonitis is the most common form of peritonitis [2,17]. Consistent with this, this study revealed that among patients without HT, CKD, or those undergoing dialysis, secondary peritonitis was the most frequently diagnosed form. Comorbidities such as HT can lead to renal damage and subsequent development of CKD [12]. In a study examining variation in PDrP outcomes, which included six countries in 2021, cardiovascular disease was the most commonly reported comorbidity [18]. Another study investigating 181 patients diagnosed with PDrP reported that 80% of patients had HT [12]. Additionally, in a study involving 2924 patients investigating peritonitis rates, HT was reported as an independent risk factor for PDrP [19]. Similarly, in our study, patients with HT, CKD, and those undergoing dialysis were most frequently found in the group with PDrP. Microorganisms can potentially reach the peritoneal cavity through catheters used in PD [4]. Although rarely, PDrP can occasionally result in hematogenous spread from other foci, primarily from the digestive and urogenital systems. However, contamination with Gram-positive microorganisms from the skin flora during dialysis exchanges is the most common cause of PDrP [2]. Previous studies in the literature have consistently reported that Gram-positive cocci are the predominant pathogens in PDrP [12,13,20,21]. On the other hand, Gram-negative aerobic and anaerobic bacteria are primarily associated with gastroduodenal perforation and, notably, in peritonitis related to intestinal or colonic sources. However, Gram-positive bacteria comprise approximately 30-40% of isolates, regardless of the perforation site [22]. In a recent multicenter epidemiological study on ıntraablominal infection in ICU patients, Gram-negative were the most common pathogens with *Enterobacterales spp*, at 64%, and *E. coli* at 45% [23]. In a retrospective study involving 414 patients diagnosed with secondary peritonitis, Gram-negative growth predominated, with *E. coli* accounting for 39% and *Enterobacteriaceae* for 24% of cases [8]. Similarly, in our study, Gram-positive growth was predominant in the PDrP group, while Gram-negative growth predominated in the secondary peritonitis group. These findings emphasize the importance of the microbiological profile in the management and treatment of peritonitis cases.

Secondary peritonitis is an inflammatory response occurring in the abdominal cavity due to perforation of hollow organs, often resulting in severe sepsis accompanied by organ failure [6]. Inflammation is typically accompanied by an elevation in the levels of acute phase reactants in serum, such as CRP and sedimentation rate [24]. Management of secondary peritonitis commonly involves addressing sepsis early on, with a focus on antibiotic therapy, fluid resuscitation, and source control [6]. Previous studies have reported a higher likelihood of sepsis development and a worse prognosis in secondary peritonitis compared to other types of peritonitis [2,11]. The observation of higher levels of CRP and sedimentation rate in secondary peritonitis in this study may be associated with this condition.

Limitations

The retrospective nature of the study and the limited patient sample from a single center inherently introduce selection bias, indicating that our results might not entirely reflect the broader population of patients with peritonitis. Furthermore, due to the lack of information regarding patients' previous hospitalizations and antibiotic usage, distinctions between community-acquired peritonitis, healthcare-associated, or nosocomial peritonitis could not be made in cases of SBP and secondary peritonitis. Additionally, the study did not investigate the etiology of secondary bacterial peritonitis.

## Conclusions

Bacterial peritonitis represents a critical and potentially life-threatening infection with systemic consequences. The timely identification of individuals at heightened risk and the implementation of preventative measures are imperative in managing this condition effectively. The distinct characteristics observed across different types of peritonitis underscore the importance of tailored approaches to diagnosis and treatment. Parameters such as CRP levels, sedimentation rates, and patient age could serve as valuable indicators in discerning between various types of peritonitis. When selecting empirical antibiotic therapy, it's crucial to consider coverage for Gram-positive pathogens in cases of peritoneal dialysis-related peritonitis and Gram-negative pathogens in secondary peritonitis.

## References

[1] Rathod S (2022). T cells in the peritoneum. Int Rev Cell Mol Biol.

[2] Pörner D, Von Vietinghoff S, Nattermann J, Strassburg CP, Lutz P (2021). Advances in the pharmacological management of bacterial peritonitis. Expert Opin Pharmacother.

[3] Laroche M, Harding G (1998). Primary and secondary peritonitis: an update. Eur J Clin Microbiol Infect Dis.

[4] Li PK, Chow KM, Cho Y (2022). ISPD peritonitis guideline recommendations: 2022 update on prevention and treatment. Perit Dial Int.

[5] Biggins SW, Angeli P, Garcia-Tsao G (2021). Diagnosis, evaluation, and management of ascites, spontaneous bacterial peritonitis and hepatorenal syndrome: 2021 practice guidance by the American Association for the Study of Liver Diseases. Hepatology.

[6] Clements TW, Tolonen M, Ball CG, Kirkpatrick AW (2021). Secondary Peritonitis and Intra-Abdominal Sepsis: An Increasingly Global Disease in Search of Better Systemic Therapies. Scand J Surg.

[7] Sahlawi MA, Wilson G, Stallard B (2020). Peritoneal dialysis-associated peritonitis outcomes reported in trials and observational studies: A systematic review. Perit Dial Int.

[8] Grotelüschen R, Heidelmann LM, Lütgehetmann M (2020). Antibiotic sensitivity in correlation to the origin of secondary peritonitis: a single center analysis. Sci Rep.

[9] Mattos AA, Wiltgen D, Jotz RF, Dornelles CM, Fernandes MV, Mattos ÂZ (2020). Spontaneous bacterial peritonitis and extraperitoneal infections in patients with cirrhosis. Ann Hepatol.

[10] Perl J, Fuller DS, Bieber BA (2020). Peritoneal dialysis-related infection rates and outcomes: results from the peritoneal dialysis Outcomes and Practice Patterns Study (PDOPPS). Am J Kidney Dis.

[11] Sartelli M, Chichom-Mefire A, Labricciosa FM (2017). The management of intra-abdominal infections from a global perspective: 2017 WSES guidelines for management of intra-abdominal infections. World J Emerg Surg.

[12] Whitty R, Bargman JM, Kiss A, Dresser L, Lui P (2017). Residual kidney function and peritoneal dialysis-associated peritonitis treatment outcomes. Clin J Am Soc Nephrol.

[13] Dzekova-Vidimliski P, Nikolov IG, Gjorgjievski N (2021). Peritoneal dialysis-related peritonitis: rate, clinical outcomes and patient survival. Pril (Makedon Akad Nauk Umet Odd Med Nauki).

[14] (2024). Peritoneal dialysis. https://www.niddk.nih.gov/health-information/kidney-disease/kidney-failure/peritoneal-dialysis.

[15] Deacon E, Canney M, McCormick B, Ramsay T, Biyani M, Brown PA, Zimmerman D (2023). The association between serum vancomycin level and clinical outcome in patients with peritoneal dialysis associated peritonitis. Kidney Int Rep.

[16] Ma X, Shi Y, Tao M (2020). Analysis of risk factors and outcome in peritoneal dialysis patients with early-onset peritonitis: a multicentre, retrospective cohort study. BMJ Open.

[17] Akçakaya A (2023). Peritonitis-an overview. Bezmialem Science.

[18] Al Sahlawi M, Zhao J, McCullough K (2022). Variation in peritoneal dialysis-related peritonitis outcomes in the peritoneal dialysis Outcomes and Practice Patterns Study (PDOPPS). Am J Kidney Dis.

[19] Lim WH, Johnson DW, McDonald SP (2005). Higher rate and earlier peritonitis in Aboriginal patients compared to non-Aboriginal patients with end-stage renal failure maintained on peritoneal dialysis in Australia: analysis of ANZDATA. Nephrology (Carlton).

[20] Hsieh YP, Chang CC, Wen YK, Chiu PF, Yang Y (2014). Predictors of peritonitis and the impact of peritonitis on clinical outcomes of continuous ambulatory peritoneal dialysis patients in Taiwan--10 years' experience in a single center. Perit Dial Int.

[21] Muthucumarana K, Howson P, Crawford D, Burrows S, Swaminathan R, Irish A (2016). The relationship between presentation and the time of initial administration of antibiotics with outcomes of peritonitis in peritoneal dialysis patients: the PROMPT study. Kidney Int Rep.

[22] Bassetti M, Eckmann C, Giacobbe DR, Sartelli M, Montravers P (2020). Post-operative abdominal infections: epidemiology, operational definitions, and outcomes. Intensive Care Med.

[23] Blot S, Antonelli M, Arvaniti K (2019). Epidemiology of intra-abdominal infection and sepsis in critically ill patients: "AbSeS", a multinational observational cohort study and ESICM Trials Group Project. Intensive Care Med.

[24] Gabay C, Kushner I (1999). Acute-phase proteins and other systemic responses to inflammation. N Engl J Med.

